# Effects of Green Tea Extract Supplementation on Inflammatory Cytokines Among Postmenopausal Women with Overweight or Obesity—A Secondary Analysis of a Randomized Controlled Trial

**DOI:** 10.3390/nu18010143

**Published:** 2026-01-01

**Authors:** Anca Cunningham, Allison Gomes, Lingqiong Meng, Sue Shapses, Laura Byham-Gray, Hamed Samavat

**Affiliations:** 1 Department of Clinical and Preventive Nutrition Sciences, School of Health Professions, Rutgers University, Newark, NJ 07102, USA; ancamariacu@gmail.com (A.C.); collina2@shp.rutgers.edu (A.G.); byhamgld@shp.rutgers.edu (L.B.-G.); 2 Department of Internal Medicine, University of Arizona College of Medicine, Phoenix, AZ 85724, USA; 3 Department of Nutritional Sciences, Rutgers University, Newark, NJ 07102, USA; 4 Department of Medicine, Rutgers RWJ Medical School, New Brunswick, NJ 08901, USA

**Keywords:** catechol-O-methyltransferase, C-reactive protein, epigallocatechin gallate, green tea extract, inflammation, interleukin-6, obesity, postmenopausal

## Abstract

**Background**: Excess adiposity induces low-grade inflammation, including increased C-reactive protein (CRP), interleukin-6 (IL-6), and tumor necrosis factor-alpha (TNF-α). Green tea contains epigallocatechin gallate (EGCG), with anti-inflammatory potential. EGCG metabolism is influenced by individual variations in catechol-O-methyltransferase (*COMT*) genotypes. **Objectives:** To evaluate the effect of green tea extract (GTE) supplementation on circulating inflammatory cytokines among postmenopausal women with overweight or obesity and differing *COMT* genotypes. **Methods**: This study is a secondary analysis of a random subset (*N* = 97) from the Minnesota Green Tea Trial (MGTT), a randomized double-blinded placebo-controlled trial. The intervention was a high-dose GTE supplement (843 ± 44 mg EGCG/day) or placebo for 1 year. Serum CRP, TNF-α, and IL-6 were measured at 0, 6, and 12 months. Absolute changes in inflammatory cytokines from baseline to month 12 were evaluated using linear mixed-effects models adjusted for age, body mass index (BMI), smoking history, physical activity, and vitamin supplement use. **Results**: The changes from month 0 to month 12 were not statistically different between the groups for any of the inflammatory cytokines measured. The overall treatment effect was not statistically significant for CRP (*p* = 0.24), IL-6 (*p* = 0.59), TNF-α (*p* = 0.36), nor for the interaction between treatment group and time (all Ps > 0.40). There was no significant interaction between treatment group and *COMT* genotype for the stated markers. **Conclusions**: A high-dose GTE supplement consumed daily for one year did not significantly decrease inflammatory cytokines among postmenopausal women with overweight or obesity. The *COMT* genotype did not modify the effects of GTE supplementation on inflammatory cytokines. Future studies with a larger sample size among those at high risk of systemic inflammation are warranted.

## 1. Introduction

An overweight or obese body mass index (BMI) is a risk factor for at least 13 types of cancer, including breast cancer [1]. Adipose tissue induces metabolic deregulation, mediated by pro-inflammatory cytokines [2,3,4,5,6]. The low-grade inflammation is characterized by an increase in circulating C-reactive protein (CRP), tumor necrosis factor-alpha (TNF-α), and interleukin-6 (IL-6) [5,7,8]. In a normal physiological state, the white adipose tissue (WAT) present in the body secretes adipokines with anti-inflammatory effects [9,10]. In the presence of increased adiposity, the enlarged adipocytes undergo hypoxia, and WAT switches to the production of cytokines, promoters of inflammation [5,9,10]. Hypoxia and cellular senescence are the main drivers of stress manifested at the endoplasmic reticulum, producing reactive oxygen species, which are free radicals that may contribute to oncogenesis [11,12]. Excess adiposity induces cellular-level adipose expansion, hypoxia, and macrophage recruitment, resulting in a persistent low-grade increase in circulating serum cytokine levels, notably CRP, IL-6, and TNF-α, which are some of the key markers of chronic inflammation [2,5]. Prolonged exposure to inflammatory cytokines results in proliferative signaling in mammary epithelium, due to aromatase upregulation, which increases estrogen production that fuels hormone-positive oncogenesis [9,13,14]. Independent of the adipose-derived estrogen fuel for tumor growth, a separate process is the systemic metabolic dysfunction present in obesity, which leads to hyperinsulinemia and increased bioavailable insulin-like growth factors (IGFs). IGF signaling activates enzymes (kinases) implicated in cell survival, notably phosphoinositide 3-kinase (PI3K)/AKT signaling and mitogen-activated protein kinases (MAPK) pathways in mammary cells, enhancing proliferation, inhibiting apoptosis, acting in sync with inflammatory cytokines to favor a milieu that promotes malignant cell proliferation [15,16]. Finally, inflammation remodels the mammary tissue microenvironment toward immunosuppressive and tumor-promoting characteristics: increased macrophages, myeloid-derived suppressor cells, and altered T-cell function [4,6]. Concurrently, chronic inflammation drives fibroblast activation and extracellular matrix remodeling, which facilitates angiogenesis. These changes allow the malignant cells to evade clearance and, therefore, tumor growth continues [4,5].

The American Cancer Society (ACS) attributes 11% of cancers in women to excess body weight [17]. Breast cancer is the most common cancer in women worldwide [18]. Increased body adiposity before menopause may be protective against breast cancer due to disruptions in ovarian function that result in decreased ovulation [13]. Pre-menopause, estrogen is synthesized in the ovaries primarily as estradiol (E2); however, after menopause, estrogen is synthesized in the peripheral tissues mainly as estrone (E1), and excess adiposity results in a greater estrogen biosynthesis, therefore a greater risk for oncogenesis in hormone-responsive tissues [9,13,14]. The Women’s Health Initiative found that each 1-unit (kg/m^2^) increase in BMI corresponds to a 3–5% increase in risk of breast cancer among postmenopausal women [19].

Green tea has been evaluated for its potential to decrease the circulating concentrations of inflammatory cytokines such as CRP, IL-6, and TNF-α [20,21,22]. Green tea contains catechins, bioactive substances that may confer anti-inflammatory activity [15,16,23,24,25]. The main catechin in green tea is epigallocatechin gallate (EGCG), a phenolic compound with antioxidant properties [15,16]. EGCG has been studied in cell lines, animal studies, and clinical trials for its potential to combat oxidative damage and to inhibit tumorigenesis [15,23]. The anti-carcinogenic effects of EGCG are exerted via several mechanisms of action: it induces cancer cell apoptosis, inhibits glycolysis, and suppresses signaling pathways involved in oncogenesis, such as nuclear factor kappa-light-chain-enhancer of activated B cells (NF-kB pathway), a regulator of inflammatory cytokines, as well as MAPK activation, and Toll-like receptor 4 (TLR4 expression) induced by inflammatory stimuli [15,16]. Moreover, green tea decreases the production of reactive oxygen species (ROS) [26,27].

The primary enzyme involved in the metabolism of EGCG is catechol-O-methyltransferase (COMT) [28]. The COMT gene is characterized by a common single nucleotide polymorphism (SNP rs4680) at nucleotide 158, with either a guanine (*G*) allele or an adenine (*A*) allele [29,30]. It has been suggested that individuals who carry the A allele (homozygous *A*/*A* or heterozygous *A*/*G*) exhibit a slow methylation rate, while carriers of two G alleles (homozygous *G*/*G*) may display a higher rate of methylation of EGCG [29,30]. A slower methylation rate may provide longer cell exposure to EGCG, which may potentially confer a greater anti-inflammatory benefit. The studies to date regarding the potential of EGCG to decrease inflammatory cytokines tested doses between 450 and 500 mg of EGCG daily, and durations of 1 to 2 months, and the findings are inconsistent [20,21,22,31].

The primary aim of this study was to evaluate the effects of daily consumption of a high-dose GTE supplement for one year on circulating levels of CRP, TNF-α, and IL-6 compared to placebo among postmenopausal women with overweight or obesity in a subset of the participants from the Minnesota Green Tea Trial (MGTT) [32]. We hypothesized that consuming a GTE supplement containing 843 mg EGCG daily for one year would decrease the serum concentrations of CRP, TNF-α, and IL-6, compared to a placebo. The secondary aim was to evaluate whether the response to treatment differs by *COMT* genotype: we hypothesized that individuals with a low or intermediate activity *COMT* genotype would have greater reductions in levels of cytokines compared to individuals with a high-activity *COMT* genotype. This study fills the gap in the literature by testing a higher dose and a longer duration than in the studies published so far; it also brings a novel approach to evaluating the effects of EGCG by considering differences in the *COMT* genotype, which previous studies have not done.

## 2. Materials and Methods

The present study is a secondary analysis of the data from the MGTT [32]. The MGTT was a randomized, placebo-controlled, double-blinded study conducted from 2009 to 2014, which enrolled 1075 postmenopausal women, aged 50–70 years, at high risk for breast cancer as determined by high breast density, calculated using screening mammogram images. The participants were generally healthy, predominantly White non-Hispanic (>95% of participants), college-educated (>90% of participants), and the majority were parous (>75% of participants), with the mean baseline BMI 25.2 (3.7) kg/m^2^, as described previously [32]. Postmenopausal status was confirmed by the absence of menses for 1 year or serum follicle-stimulating hormone (FSH) > 23 mIU/mL [33].

The exclusion criteria were: regular consumption of green tea (>1 cup per week), use of hormone therapy within the six months before enrollment, active smoking status at the time of enrollment, consumption of more than 7 alcoholic drinks a week, presence of breast implants, concurrent participation in a weight loss program, weight changes of more than 10 pounds within the previous year, or BMI outside of the range 19–40 kg/m^2^. Participants were also excluded if they had any chronic disease (e.g., liver disease, rheumatoid arthritis, diabetes mellitus, and others) other than controlled hypertension, or use of certain medications (i.e., Methotrexate or Enbrel). Participants were randomized to a GTE supplement or placebo group and stratified by COMT genotype. Randomization was conducted using the computer-generated permuted block scheme with a block size of 8 stratified by COMT genotype, by the Investigational Drug Services pharmacy at the University of Minnesota Medical Center–Fairview. All study staff and participants were blinded to the random allocation sequence, with only the pharmacy staff aware of the assignments. This trial was registered on 8 June 2009, at clinicaltrials.gov as NCT00917735, where it can be accessed. No important changes were made to the trial protocol after it commenced, and all outcomes and analyses were conducted as prespecified. However, several ancillary studies have been conducted to explore additional outcomes, including body composition, blood lipids, and appetite-related hormones.

The intervention consisted of a decaffeinated GTE supplement (Corban complex GTB; Investigational New Drug #103,431) delivering a total daily dose of 843.0 ± 44.0 mg of EGCG in the four capsules. The total amount of catechins consumed per day was 1315.0 ± 80.0 mg, including epigallocatechin (EGC), catechin, epicatechin (EC), EGCG, gallocatechin gallate (GCG), epicatechin gallate (ECG), catechin gallate (CG), and gallocatechin (GC). The placebo capsules were identical looking to the GTE capsules, and contained a combination of maltodextrin (50%), cellulose (49.5%), and magnesium stearate (0.5%) [32]. The participants were instructed to take four capsules daily: two with breakfast and two with dinner. Adherence to the supplement intake was established in the original study, primarily through capsule count, with excellent compliance (96.5% of prescribed capsules) [32]. For the present subset, the average compliance rate was 96.1%, slightly lower than the parent trial.

The anthropometric data and vital signs were collected and recorded by the trained nursing staff at multiple time points during the trial. Two questionnaires were utilized: the Health History Questionnaire (HHQ)—developed by the investigators of the MGTT and administered at baseline, and the Dietary History Questionnaire, version I (DHQ I) [34]—a validated food frequency questionnaire (FFQ), developed by the National Cancer Institute (NCI), administered at baseline and end of the study, to estimate daily dietary intake over the previous year based on participant self-reported consumption of selected foods [35]. The raw dietary data were analyzed using NCI Diet*Calc software version 1.4.3 (a public domain tool created by the U.S. government) [36].

All adverse events were monitored and recorded by study staff throughout the intervention period, and interim analyses and stopping rules have been described previously [32]. There were no significant differences in the percentages of participants with adverse events between the GTE and placebo groups in the MGTT. The present study was approved by the Internal Review Boards of the University of Minnesota and Rutgers University. Written informed consent was obtained from all participants prior to enrollment in the MGTT, in accordance with the Declaration of Helsinki.

**Figure 1:**
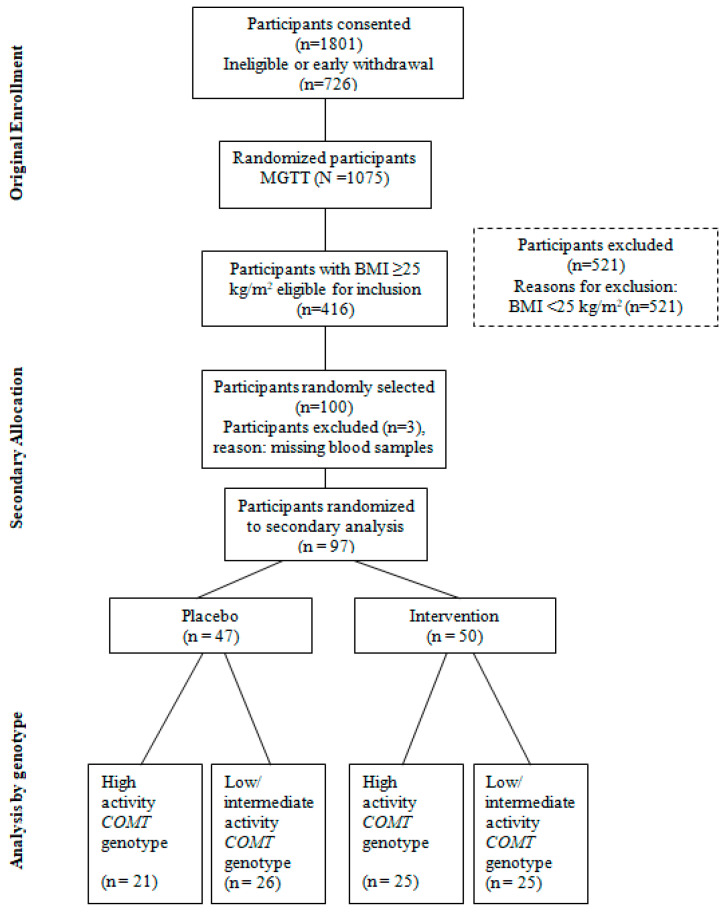
Flow diagram showing the selection of a subset of the MGTT participants.

The present secondary analysis expanded the scope of the MGTT in a randomly selected subset (*N* = 97) of participants with overweight and obese BMI (Figure 1), to determine the effects of GTE consumption daily for one year on selected inflammatory cytokines: CRP, TNF-α, and IL-6. The selected inflammatory cytokines were measured in fasting blood samples (minimum of 10 h fast), collected and processed by trained staff at three time points: baseline (month 0), 6 months, and 12 months, and stored in a −80 °C freezer until the later time when the analysis was performed.

Circulating inflammatory cytokines, IL-6, CRP, and TNF-alpha were measured by a single technician blinded to the intervention status and analyzed by the ELISA method (kits provided by R&D Systems, Minneapolis, MN, USA: D6050, DCRP00, and DTA00D, respectively). Two quality control samples were included in each batch of samples, and all samples were analyzed in singlicate. All three samples from a given subject were measured in the same analytic run to reduce interassay variability. The interassay coefficients of variation (CVs) were 3.3% for CRP, 7.1% for IL-6, and 5.3% for TNF-α, respectively.

For the primary hypothesis, the data were missing for 21 subjects for IL-6 at baseline and 18 subjects at 12 months, and 3 subjects for TNF-α at baseline due to values being undetectable and below the assay threshold. Patterns of missing data were assessed with the Little MCAS test, which suggested that the data were consistent with being missing completely at random (χ^2^(5) = 10.41, *p* = 0.064). The independent variable was the treatment, with the two possible groups of GTE or placebo. The dependent variables or outcomes of interest were circulating concentrations of CRP, TNF-α, and IL-6 at baseline, 6 months, and 12 months. The pre-specified covariates were age, BMI, smoking history, physical activity, and use of dietary supplements, as potential factors affecting inflammation [17,19]. Bivariate analyses were conducted between baseline demographics, lifestyle, dietary characteristics, primary circulating estrogens including estrone and estradiol, and the outcome variables, and no additional covariates were identified.

An a priori power analysis was conducted, and the present study possessed at least 80% statistical power to detect 10% and 17% reduction in CRP and IL-6 levels, respectively, in the GTE-treated versus placebo-controlled women with a sample size of 50 per arm (*N* = 100 in total), assuming a two-sided significance level of 0.05. While the performed power calculation applies to the primary aim of this proposed project, a power analysis was not performed for the secondary aim, which is intended to be exploratory in nature.

The inflammatory cytokines data were analyzed for normality via visual inspection using histograms, box-and-whisker plots, and Q-Q plots. Given that the data were not normally distributed, natural log-transformation was used to normalize the distribution of serum levels of CRP, IL-6, and TNF-α at baseline, month 6, and month 12, and results were presented as geometric means (95% CIs). To test the primary hypothesis, a single linear mixed-effects model (LMM) was built for each outcome with both a fixed effect model (for the main effects and interactions) and a random effect for subject to account for multiple correlated measurements within each subject. Absolute changes were calculated from baseline to 12 months and reported as arithmetic means (95% CIs). To test the primary hypothesis, changes were evaluated between the treatment groups using an LMM model, adjusted for the pre-specified covariates as well as for baseline values of the corresponding cytokines. To test the secondary hypothesis, LMM models examined the overall treatment effects and the interaction between treatment and *COMT* genotype on inflammatory cytokines, adjusting for the baseline values and pre-specified covariates.

The present statistical analysis was reported as intent-to-treat; however, per-protocol analysis was also conducted, and there were no statistical differences in the findings. The data analysis was performed with IBM SPSS version 29.0 (IBM Corp, Armonk, NY, USA) and SAS version 9.4 (SAS Institute, Inc., Cary, NC, USA). *p* values <0.05 were considered statistically significant.

## 3. Results

The baseline characteristics and dietary components data by treatment group are shown in Table 1 and Table 2, respectively.

Overall, participants had a mean (±SD) age of 60.4 ± 5.3 years at baseline. The mean (±SD) BMI value of the participants was 28.9 (±3.0) kg/m^2^, and 61% of BMI values were in the overweight range, while 39% in the obesity range. Their median (IQR) intake of alcohol was reported as 2.6 (0.8, 7.8)g/day. As far as past smoking history, 74% of the participants never smoked. Most participants (90%) reported using dietary supplements. The median (IQR) physical activity was 29.0 (12.0, 50.0) metabolic equivalent of task (MET) hours per week. Results from bivariate analyses showed that neither baseline characteristics nor dietary intake data at baseline or 12 months were statistically significantly different between the treatment groups.

### 3.1. Treatment Effect on Inflammatory Cytokines

Bivariate analyses between baseline demographics, lifestyle, and dietary characteristics and outcomes showed a weak positive correlation between BMI and CRP (rho = 0.380, *p* < 0.01) and IL-6 (rho = 0.328, *p* = 0.004). No other bivariate analysis was significant—therefore, only the pre-specified covariates (age, BMI, smoking history, physical activity, and dietary supplement use) were included in the LMM model. There were no statistically significant differences in the circulating levels of CRP, IL-6, and TNF-α at 0, 6, and 12 months between the treatment groups (Table 3). Additionally, when changes from baseline to month 12 for inflammatory cytokines were compared between the treatment groups, the findings were not statistically significantly different, based on the LMM model adjusted for baseline values and the pre-specified covariates. Similarly, there was no overall treatment effect for CRP (*p* = 0.240), IL-6 (*p* = 0.589), and TNF-α (*p* = 0.361). The interaction between treatment and time was not statistically significant for any of the inflammatory cytokines.

### 3.2. Impact of COMT Genotype Differences

This study also explored whether differences related to the *COMT* genotype, dichotomized as high activity genotype (*G*/*G*, wild type) and the low/intermediate activity genotype (*A*/*A*) or (*A*/*G*), modulate the response to treatment, or affect changes over time (Table 4).

Based on the results from the LMM model, adjusted for the pre-specified covariates, there were no statistically significant differences in the levels of CRP, IL-6, and TNF-α between the groups at any time point within each *COMT* genotype activity, except that TNF-α levels were significantly higher in the GTE than in the placebo group at baseline within the high-activity *COMT* genotype (*p* = 0.049). Changes from baseline to 12 months in the outcomes were not different between the GTE and the placebo groups within the high-activity (*G*/*G*, wild type) or the low/intermediate activity (*A*/*A* or *A*/*G*)*COMT* genotype. There was no statistically significant interaction between treatment and *COMT* genotype for CRP, IL-6, and TNF-α.

## 4. Discussion

The purpose of the MGTT was to evaluate the effects of a high-dose, long-term GTE supplement on biomarkers of breast cancer risk among postmenopausal women. The present secondary analysis aimed to investigate the effects of the stated supplement on inflammatory cytokines in a subset of participants with overweight or obesity, randomly selected from the MGTT parent trial. The primary hypothesis was that after 12 months of supplementation with a high-dose GTE that delivered 843 ± 44 mg EGCG per day, there would be a significant reduction in the inflammatory cytokines CRP, IL-6, and TNF-α. A secondary hypothesis was that the *COMT* genotype would impact response to treatment, specifically that there would be a greater decrease in inflammatory cytokines in the group with a low/intermediate *COMT* genotype (alleles *A*/*A*, *A*/*G*) as compared to the group with a high activity *COMT*(alleles *G*/*G*). This study did not find a significant decrease in inflammatory cytokines in response to a high-dose GTE consumed daily for one year. The *COMT* genotype did not modify the effects of GTE supplementation on inflammatory cytokines.

The baseline values of inflammatory cytokines in our sample are suggestive of the absence of inflammation, which may limit the ability to detect significant changes in these markers. The elusive nature of low-grade inflammation has made normative ranges difficult to establish [7,37,38]. For CRP, there is an interpretative guideline for the range 3–10 mg/L as indicative of low-grade inflammation, while values <3 mg/L, which is the mean value of our sample, is indicative of the absence of inflammation [39]. For IL-6 and TNF-α, a recent study by Meng et al., which included only postmenopausal women, reported that the mean (SD) values for healthy controls were 2.1 (2.0) pg/mL and 12.1 (13.4) pg/mL, respectively [40]. In the present study, baseline values were IL-6 <2 pg/mL and TNF-α <10 pg/mL, suggestive of the absence of inflammation or low-grade inflammation.

Our findings add to the literature published to date on the effects of GTE supplements on inflammatory cytokines. Unlike the present study, which showed null findings, the only other study that used a similar sample of exclusively postmenopausal women with obesity (BMI 30–35 kg/m^2^) enrolled 28 participants, tested a lower dose of 449 mg EGCG/day for 60 days, and found a significant reduction in serum CRP [21]. While three other RCTs assessed the impact of GTE on biomarkers of inflammation among women, their samples differed in age and BMI ranges, menopausal status, and the dose and duration of GTE used compared to the current study [20,22,31]. Of these studies, only the study by Noronha et al. [20] showed a significant change in IL-6 (a decrease of 12.7% from baseline) after taking 450 mg EGCG for 8 weeks [20]. This RCT included 63 women, with a mean age of 36.2 ± 9.1 years and a mean BMI of 48 ± 9.3 kg/m^2^, of whom 21 participants had BMI values in the normal range, and 42 had severe obesity [20]. The other two RCTs also included younger women than the present study (mean ages 41.1 ± 8.4 years [31] and 23.2 ± 5.2 years [22]. One study included participants with a BMI range of 30.0–39.9 kg/m^2^ [31], while the other study included women with polycystic ovarian syndrome (PCOS), both with normal weight and with overweight or obese BMI, ranging from 20 to 35 kg/m^2^ [22]. The participants in both RCTs received the same dose of GTE (500 mg EGCG daily), although the length of intervention varied from 4 weeks [31] to 45 days [22]. Results from these two studies showed no significant changes in any of the inflammatory markers [22,31].

As far as the impact of the *COMT* genotype on the response to treatment, it needs to be noted that the sample in this study was divided into four subgroups, and we may not have had sufficient power to detect the effect of the *COMT* genotype on any of the studied inflammatory markers. Regarding the impact of the *COMT* genotype on the study endpoints, we observed a significant difference in TNF-α levels between the treatment groups at baseline within the high activity *COMT* genotype.

The systemic chronic inflammation present in states of increased body adiposity has been characterized by slight elevations of the serum cytokines [5]. Therefore, the amount of change that may be observed is also smaller than in other pathophysiology that involves a greater burden of inflammation. From a clinical but not statistical significance perspective, it is notable that the impact of GTE supplementation was not null, and the group receiving the GTE supplement sustained slight non-significant decreases in inflammatory cytokines, while the placebo group experienced slight non-significant increases in the same inflammatory cytokines (Supplemental Figures S1 and S2). Based on these findings, it is intriguing to speculate that in the absence of any intervention, as in the placebo group, there may be a tendency towards subtle increases in inflammatory cytokines over time, in the presence of persistent increased body adiposity. The present study is the second study that evaluated GTE supplements in a sample of exclusively postmenopausal women with an overweight or obese BMI. The other study that enrolled a sample from the same demographic subset of postmenopausal women with overweight or obesity only measured CRP and showed a statistically significant reduction in this marker in the intervention group compared to placebo. The pre- and post-CRP levels in both groups were <1 mg/L; therefore, the small magnitude of change may not provide clinical benefits if baseline inflammation was not present, even if the intergroup comparison was statistically significant [21].

Furthermore, it needs to be stated that EGCG has a limited bioavailability, poor intestinal absorption, and undergoes extensive phase-II metabolism (including COMT methylation reactions), which results in a substantial decrease in systemic and tissue exposures; therefore, escalating the dose may not result in an increased response [15,26]. Although the current evidence is inconclusive, broader pharmacokinetic evidence suggests that prolonged GTE supplementation may induce metabolic adaptation, with upregulation of conjugating enzymes leading to a plateau effect in systemic levels despite dose escalation [15,26,27]. As far as safety concerns, some clinical trials have reported statistically significant increases in serum transaminases at high doses of EGCG supplement [41,42]. As the dose used in our trial was a high dose in the upper range of what is considered safe, dose escalation in future trials is not advised [23,26]. Our null results should be interpreted with caution, in view of the above-described pharmacodynamics, and considering that serum cytokines do not capture tissue response, such as local response in the adipose or mammary tissue [23,26]. The present study aimed to investigate the impact of GTE intake on systemic low-grade inflammation in the presence of increased body adiposity, and specific tissue response was beyond the scope of this study.

This study has some limitations and strengths. As a secondary analysis of data from a large clinic trial designed for different primary endpoints, the present study may lack specificity and sensitivity in measuring the effects of GTE on inflammatory cytokines. Although one year may be considered a long duration for an RCT, it may be insufficient to observe significant changes in chronic inflammatory markers, particularly in a population with low baseline inflammation status. A further limitation is that cytokines were measured in singlicate. While duplicate measurements can enhance analytical precision, we employed high-sensitivity assays with rigorous internal controls to mitigate variability and maintain data quality. In addition, all inter-assay CVs remained within the manufacturer’s acceptable range (below 7.5%), suggesting that singlicate measurements provided sufficient reliability for this analysis. Based on *a priori* power calculations, the present study had adequate power to capture significant changes for the primary endpoints; however, it was based on a medium effect size, and the sample size may still be relatively small for detecting subtle differences. The present study derives a strong internal validity from the parent study, which was a randomized, double-blinded, placebo-controlled trial. Standardized protocols were used by trained staff for measuring and collecting the data, and the tools for collection included a validated food frequency questionnaire. The laboratory analysis of the serum markers was conducted in accordance with high industry standards. The dose and duration of the study are other strengths of the present study, as 12 months is a longer duration, and 843 ± 44 mg EGCG is the highest dose studied in the literature to date [20,21,22,31]. The external validity of this study is limited to the population described: postmenopausal women, aged 50–70 years, predominantly White, generally healthy, physically active, with overweight or obesity, living in the Midwest area of the US. Therefore, the findings may not be generalized beyond the population described.

## 5. Conclusions

This secondary analysis found that long-term, high-dose GTE supplementation (EGCG 843 mg/day for one year) does not reduce systemic inflammation in postmenopausal women with overweight or obesity, particularly those without elevated inflammation at baseline. The *COMT* genotype did not modify the response to treatment. Postmenopausal women continue to be a demographic subset that is underrepresented in the literature on this topic, as evidenced by only one other study conducted to date to examine the same hypothesis as this study [21]. The present study is the first to evaluate the impact of the *COMT* genotype on inflammatory cytokines in response to a GTE supplement. Although no statistically significant results were found, the study is important because it addresses pharmacogenetic modulation, helping refine expectations about GTE supplements and anti-inflammatory agents in low-risk populations. Future studies with more racially and ethnically diverse samples, larger samples, and purposive samples, among those at high risk of systemic inflammation, are warranted. The general population in the US engages in the use of dietary supplements, reportedly as many as 75% of the adult population in the US used dietary supplements in 2024 [43]. Therefore, evidence-based guidelines are needed to guide consumer choice and prevent misuse.

## Figures and Tables

**Table 1:** Baseline characteristics of study participants by treatment group.

Characteristic	GTE (*n* = 50)	Placebo (*n* = 47)
Age (years) ^1^	60.4 ± 5.2	60.4 ± 5.5
BMI (kg/m^2^) ^1^	29.1 ± 3.1	28.7 ± 2.7
Alcohol (g/day) ^2^	2.6 (0.9, 7.6)	3.4 (0.8, 10.8)
Physical activity (MET-h/week) ^2^	40.3 (12.0, 58.0)	24.5 (12.0, 40.5)
Smoking status, *n* (%)		
Never	34 (68.0)	38 (83.0)
Former	16 (32.0)	9 (17.0)
Vitamin supplement use, *n* (%)		
No	5 (10.0)	5 (11.0)
Yes	45 (90)	42 (89.0)
*COMT* genotype, *n* (%)		
Low/intermediate activity (*A*/*A*)/(*A*/*G*)	25 (50.0)	26 (55.0)
High activity (*G*/*G*)	25 (50.0)	21 (45.0)

Abbreviations: BMI, body mass index; GTE, green tea extract; MET, metabolic equivalent of task. ^1^ Reported as Mean ± standard deviation. ^2^ Reported as median (interquartile range).

**Table 2:** Dietary intake by treatment group at baseline.

Component	GTE ^1^ (*n* = 50)	Placebo ^1^ (*n* = 47)
**Energy (kcals/day)**		
Baseline	1380.9 (977.3, 1993,6)	1376.4 (1108.2, 1752.2)
12 months	1404.6 (833.3, 1847.8)	1220.3 (1004.3, 1602.1)
**Protein (% kcals)**		
Baseline	16.3 (14.1, 18.0)	16.2 (15.0, 17.8)
12 months	16.1 (14.6, 18.0)	16.6 (14.9, 19.1)
**Carbohydrate (% kcals)**		
Baseline	48.6 (43.7, 56.6)	52.2 (46.6, 55.3)
12 months	48.2 (43.0, 52.9)	51.3 (44.7, 55.4)
**Fat (% kcals)**		
Baseline	33.2 (28.6, 36.9)	31.4 (27.7, 35.5)
12 months	35.7 (31.1, 39.8)	31.2 (27.6, 36.5)
**Saturated fat (% kcals)**		
Baseline	10.2 (9.5, 12.3)	10.2 (8.8, 11.2)
12 months	11.6 (9.4, 12.8)	10.6 (8.8, 12.7)
**Monounsaturated fat (% kcals)**		
Baseline	12.7 (10.4, 14.6)	11.9 (10.6, 13.2)
12 months	13.5 (11.7, 15.4)	12.2 (10.3, 14.6)
**Polyunsaturated fat (% kcals)**		
Baseline	7.1 (5.9, 9.1)	6.5 (5.5, 8.2)
12 months	7.7 (6.1, 9.4)	6.5 (5.6, 7.7)
**Total *n*-3 fatty acids (g)**		
Baseline	1.0 (0.7, 1.4)	1.0 (0.7, 1.2)
12 months	1.0 (0.6, 1.3)	0.9 (0.7, 1.2)
***Trans* fat (g)**		
Baseline	2.3 (1.8, 3.8)	2.8 (2.0, 3.6)
12 months	2.4 (1.7, 3.4)	2.2 (1.7, 3.7)
**Fiber (g)**		
Baseline	16.2 (10.9, 21.4)	15.3 (12.2, 19.0)
12 months	16.0 (9.2, 20.6)	13.4 (10.8, 19.6)
**Added sugar (% kcals)**		
Baseline	9.6 (7.2, 12.6)	10.0 (8.2, 13.7)
12 months	9.2 (7.6, 11.2)	9.7 (7.6, 12.7)

Abbreviations: GTE, green tea extract; kcals, kilocalories. Values shown as median (interquartile range). ^1^ Placebo (*n* = 46) at baseline for energy, protein, carbohydrate, fat, saturated fat, monounsaturated fat, polyunsaturated fat, and added sugar, and at 12 months for all dietary data. GTE (*n* = 47) at 12 months for energy, protein, carbohydrate, fat, saturated fat, monounsaturated fat, polyunsaturated fat, and added sugar, and GTE (*n* = 49) at 12 months for total *n*-3 fatty acids, *trans* fat, and fiber.

**Table 3:** Geometric means (95% CI) of inflammatory cytokines, by treatment group.

Inflammatory Cytokines	*n*	GTE	*n*	Placebo	*p*-Value ^a^	*p*-Value ^b^	*p*-Value ^c^
**CRP (mg/L)**							
**Baseline**	50	2.03 (1.58,2.62)	47	1.76 (1.36, 2.29)	0.44	0.24	0.97
**Month 6**	48	2.00 (1.54, 2.60)	44	1.79 (1.36, 2.34)	0.43		
**Month 12**	50	2.09 (1.62, 2.70)	47	1.92 (1.48, 2.50)	0.65		
**Δ 0–12 m**	50	−0.17 (−0.66, 0.31)	47	0.31 (−0.52, 1.15)	0.37		
**IL-6 (pg/mL)**							
**Baseline**	38	1.88 (1.32, 2.66)	38	1.36 (0.96, 1.93)	0.24	0.59	0.43
**Month 6**	38	1.85 (1.30, 2.62)	35	1.95 (1.35, 2.80)	0.95		
**Month 12**	36	2.11 (1.47, 3.02)	43	2.00 (1.44, 2.78)	0.77		
**Δ 0–12 m**	30	−0.65 (−1.73, 0.43)	36	0.69 (−0.29, 1.67)	0.24		
**TNF-α (pg/mL)**							
**Baseline**	49	8.99 (7.85, 10.29)	45	9.01 (7.82, 10.37)	0.89	0.36	0.70
**Month 6**	48	9.16 (7.99, 10.50)	44	9.36 (8.12, 10.80)	0.85		
**Month 12**	50	8.36 (7.32, 9.56)	47	9.33 (8.12, 10.71)	0.27		
**Δ 0–12 m**	49	−0.36 (−1.67, 0.95)	45	0.34 (−1.00, 1.68)	0.55		

Abbreviations: CRP, C-reactive protein; GTE, green tea extract; IL-6, interleukin-6; TNF-α, tumor necrosis factor alpha; Δ, absolute changes from month 0 to month 12. Values are reported as geometric means (95% CI) at baseline, month 6, and month 12, and arithmetic means (95% CI) for absolute changes from month 0 to month 12. ^a^ *p*-values for the difference between the GTE and placebo groups, based on a single linear mixed-effects model adjusted for age, BMI, smoking history, physical activity, and dietary supplement use at baseline. The *p*-values for comparing absolute changes between the groups were additionally adjusted for the baseline values of the inflammatory cytokines. ^b^ *p* value for overall treatment effect based on a linear mixed-effects model adjusted for age, BMI, smoking history, physical activity, and dietary supplement use at baseline. ^c^ *p* value for interaction between treatment and time based on a linear mixed-effects model adjusted for age, BMI, smoking history, physical activity, and dietary supplement use at baseline.

**Table 4:** Geometric means (95% CI) of inflammatory cytokines, by treatment group stratified by *COMT* genotype.

Inflammatory Cytokines	COMT Genotype							
	High Activity (*G*/*G*)				Low/Intermediate Activity (*A*/*A* or *G*/*A*)			
	*n*	GTE	*n*	Placebo	*n*	GTE	*n*	Placebo
**CRP (mg/L)**								
**Baseline**	25	2.17 (1.52, 3.10)	21	2.35 (1.59, 3.47)	25	1.91 (1.34, 2.73)	26	1.40 (0.98, 1.98)
**Month 6**	23	2.20 (1.51, 3.19)	20	2.27 (1.52, 3.39)	25	1.84 (1.29, 2.63)	24	1.46 (1.02, 2.11)
**Month 12**	25	2.56 (1.79, 3.66)	21	2.16 (1.47, 3.20)	25	1.71 (1.20, 2.45)	26	1.75 (1.23, 2.48)
**Δ 0–12 m**	25	0.09 (−0.26, 0.43)	21	−0.26 (−0.93, 0.41)	25	−0.44 (−0.86, −0.01)	26	0.78 (0.12, 1.43)
***p*-value ^1^**	0.671							
**IL-6 (pg/mL)**								
**Baseline**	19	2.08 (1.27, 3.41)	18	1.28 (0.77, 2.13)	19	1.70 (1.03, 2.78)	20	1.44 (0.89, 2.32)
**Month 6**	18	2.45 (1.47, 4.07)	17	2.45 (1.45, 4.14)	20	1.43 (0.88, 2.32)	18	1.57 (0.94, 2.61)
**Month 12**	19	2.33 (1.42, 3.82)	19	1.75 (1.07, 2.61)	17	1.88 (1.12, 3.17)	24	2.23 (1.43, 3.46)
**Δ 0–12 m**	16	−0.44 (−1.15, 0.28)	17	0.98 (−0.04, 2.01)	14	−0.89 (−1.91, 0.12)	19	0.43 (−0.09, 0.95)
***p*-value ^1^**	0.316							
**TNF-α (pg/mL)**								
**Baseline**	24	11.24 (9.35, 13.51)	20	8.78 (7.17, 10.73)	25	7.26 (6.06, 8.69)	25	9.20 (7.68, 11.02)
**Month 6**	23	10.94 (9.06, 13.20)	20	9.26 (7.57, 11.33)	25	7.78 (6.50, 9.33)	24	9.45 (7.86, 11.36)
**Month 12**	25	10.74 (8.97, 12.86)	21	10.35 (8.50, 12.60)	25	6.52 (5.44, 7.80)	26	8.58 (7.19, 10.23)
**Δ 0–12 m**	24	0.14 (−0.96, 1.24)	20	1.37 (0.25, 2.48)	25	−0.84 (−1.49, 0.52)	25	−0.48 (−1.49, 0.52)
***p*-value ^1^**	0.307							

Abbreviations: COMT, catechol-O-methyltransferase; CRP, C-reactive protein; GTE, green tea extract; IL-6, interleukin-6; TNF-α, tumor necrosis factor alpha; Δ, absolute changes from month 0 to month 12. Values are presented as geometric means (95% CI) at baseline, month 6, and month 12, and arithmetic means (95% CI) for absolute changes from month 0 to month 12. ^1^ *p*-value for interaction between group and *COMT* genotype, based on a linear mixed-effects model adjusted for baseline values, age, BMI, smoking history, physical activity, and dietary supplement use at baseline; arithmetic means reported for absolute changes from month 0 to month 12.

## Data Availability

The original contributions presented in this study are included in the article/Supplementary Materials. Further inquiries can be directed to the corresponding author.

## Supplementary Materials

The following supporting information can be downloaded at: https://www.mdpi.com/article/10.3390/nu18010143/s1, Figure S1: Change in inflammatory cytokines from baseline to months 6 and 12 by treatment group; Figure S2: Change in inflammatory cytokines from baseline to months 6 and 12 by treatment and COMT genotype activity groups.

## Abbreviations

The following abbreviations are used in this manuscript:

*A*	Adenine
BMI	Body mass index
CI	Confidence interval
COMT	Catechol-O-methyltransferase
CRP	C-reactive protein
EGCG	Epigallocatechin gallate
*G*	Guanine
GTE	Green tea extract
IL-6	Interleukin-6
IQR	Interquartile range
LMM	Linear mixed model
MET	Metabolic equivalent of task
MGTT	Minnesota Green Tea Trial
RCT	Randomized controlled trial
SD	Standard deviation
SNP	Single nucleotide polymorphism
TNF-α	Tumor necrosis factor alpha
