# Supplementary material for: Effects of Green Tea Extract Supplementation on Inflammatory Cytokines Among Postmenopausal Women with Overweight or Obesity—A Secondary Analysis of a Randomized Controlled Trial
Source: Nutrients. 2026 Jan 1;18(1):143. doi: 10.3390/nu18010143 (PMC12787635; doi:10.3390/nu18010143)
Supplement: Supplementary file 1 [file nutrients-18-00143-s001.zip › nutrients-4059836-supplementary.pdf]

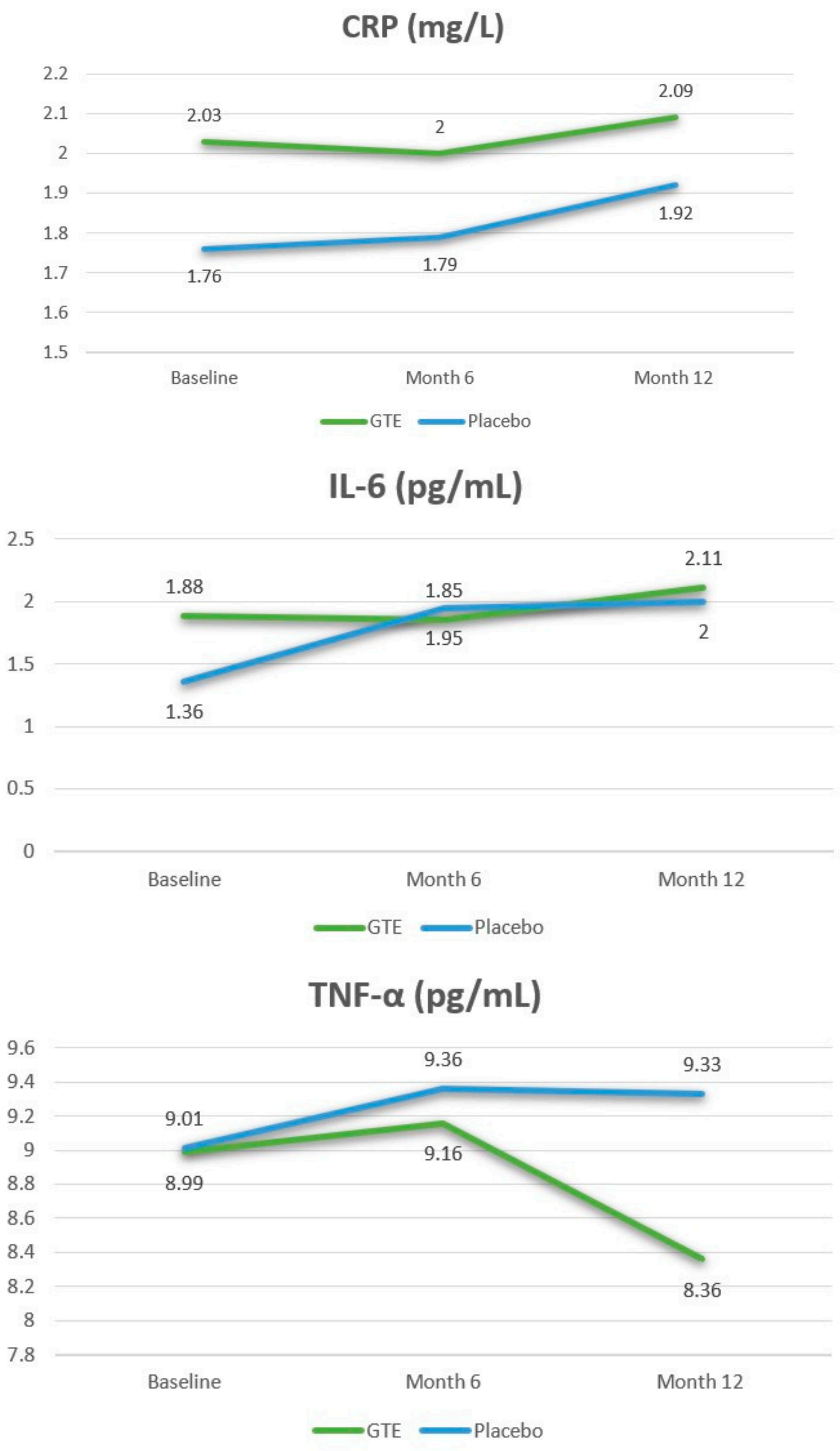

Supplemental Figure S1. Change in inflammatory cytokines from baseline to months 6 and 12 by treatment group

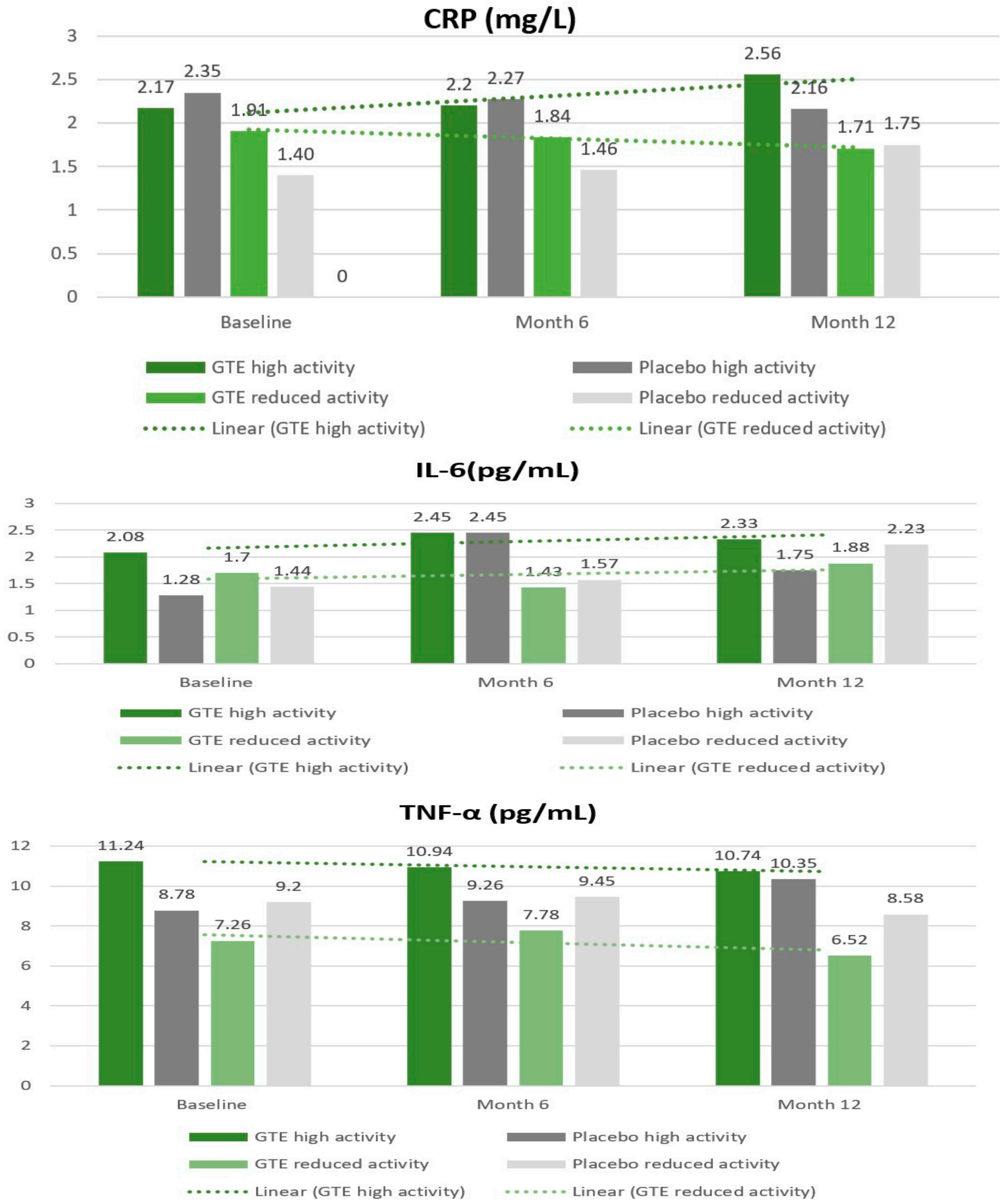

Supplemental Figure S2. Change in inflammatory cytokines from baseline to months 6 and 12 by treatment and *COMT* genotype activity groups
